# Platelet-derived microparticles stimulate the invasiveness of colorectal cancer cells via the p38MAPK-MMP-2/MMP-9 axis

**DOI:** 10.1186/s12964-023-01066-8

**Published:** 2023-03-07

**Authors:** Hassan Kassassir, Izabela Papiewska-Pająk, Jakub Kryczka, Joanna Boncela, M. Anna Kowalska

**Affiliations:** 1 Laboratory of Cellular Signaling, Institute of Medical Biology, Polish Academy of Science, Lodowa 106, Lodz, Poland; 2 The Children’s Hospital of Philadelphia, Philadelphia, PA USA

**Keywords:** Platelet-derived microparticles, Colorectal cancer, Metalloproteases

## Abstract

**Background:**

Metastasis is the main cause of death in patients with colorectal cancer (CRC). Apart from platelets, platelet-derived microparticles (PMPs) are also considered important factors that can modify the activity of cancer cells. PMPs are incorporated by cancer cells and can also serve as intracellular signalling vesicles. PMPs are believed to affect cancer cells by upregulating their invasiveness. To date, there is no evidence that such a mechanism occurs in colorectal cancer. It has been shown that platelets can stimulate metalloproteases (MMPs) expression and activity via the p38MAPK pathway in CRC cells, leading to their elevated migratory potential. This study aimed to investigate the impact of PMPs on the invasive potential of CRC cells of various phenotypes via the MMP-2, MMP-9 and p38MAPK axis.

**Methods:**

We used various CRC cell lines, including the epithelial-like HT29 and the mesenchymal-like SW480 and SW620. Confocal imaging was applied to study PMP incorporation into CRC cells. The presence of surface receptors on CRC cells after PMP uptake was evaluated by flow cytometry. Transwell and scratch wound-healing assays were used to evaluate cell migration. The level of C-X-C chemokine receptor type 4 (CXCR4), MMP-2, and MMP-9 and the phosphorylation of ERK1/2 and p38MAPK were measured by western blot. MMP activity was determined using gelatine-degradation assays, while MMP release was evaluated by ELISA.

**Results:**

We found that CRC cells could incorporate PMPs in a time-dependent manner. Moreover, PMPs could transfer platelet-specific integrins and stimulate the expression of integrins already present on tested cell lines. While mesenchymal-like cells expressed less CXCR4 than epithelial-like CRC cells, PMP uptake did not increase its intensity. No significant changes in CXCR4 level either on the surface or inside CRC cells were noticed. Levels of cellular and released MMP-2 and MMP-9 were elevated in all tested CRC cell lines after PMP uptake. PMPs increased the phosphorylation of p38MAPK but not that of ERK1/2. Inhibition of p38MAPK phosphorylation reduced the PMP-induced elevated level and release of MMP-2 and MMP-9 as well as MMP-dependent cell migration in all cell lines.

**Conclusions:**

We conclude that PMPs can fuse into both epithelial-like and mesenchymal-like CRC cells and increase their invasive potential by inducing the expression and release of MMP-2 and MMP-9 via the p38MAPK pathway, whereas CXCR4-related cell motility or the ERK1/2 pathway appears to not be affected by PMPs.

Video Abstract

**Supplementary Information:**

The online version contains supplementary material available at 10.1186/s12964-023-01066-8.

## Background

Colorectal cancer is a poorly diagnosed type of neoplasm that is still without successful treatment. The formation of metastases remains a challenge in the treatment of this cancer. The mechanism of metastasis to distant tissues/organs is more than a single-factor-dependent process and includes several steps. It has been proven that cancer growth and metastatic spread are regulated by circulating blood platelets [1]. Cancer cells can stimulate platelet activation and aggregation, and by direct interaction with cancer cells in the bloodstream, platelets can protect cancer cells from the host immune system [2], limit their exposure to shear stress, and promote their adhesion to the vessel wall and extravasation [3, 4]. Upon activation, platelets release small vesicles encapsulated by the plasma membrane called platelet-derived microparticles (PMPs). PMPs are well-established regulators of intracellular communication, and currently, much attention has been given to the potential role of PMPs in cancer progression. The abundance of PMPs has been observed in numerous types of cancerous malignancies, such as skin, lung, gastric, breast and colorectal cancers, but the mechanisms of PMP action and their role in cancer progression and metastasis are still poorly understood [5]. It has been proposed that PMPs interact with cancer cells and regulate cancer spread by promoting angiogenesis, supporting cell invasion, and potentiating metastasis [6]. PMPs can affect cancer development by delivering bioactive contents, such as proteins, nucleic acids, signalling molecules, membrane receptors or lipids, that act on recipient cells and are able to modify the intercellular signalling cascade [7]. Platelets and PMPs, which are formed from activated platelets, contain numerous receptors, including specific integrins. It has been shown that some of these integrins can be transferred by PMPs into cancer cells, thus increasing the adhesive potential of recipient cells [8]. Another example of a receptor that is present in PMPs is the chemokine receptor CXCR4. Elevated levels of CXCR4 are associated with cancer aggressiveness and survival [9, 10]. It has been proven that the transfer of CXCR4 by PMPs enhances the proliferation and migration of recipient cancer cells. However, there is a lack of data regarding whether such a mechanism can be observed in CRC. PMP-cancer cell interactions have been intensively studied, including the activation of intracellular kinases (such as mitogen-activated protein kinases, MAPKs) and the subsequent elevation of the expression/release of metalloproteases (MMPs). MMPs have been considered crucial factors in the migration and invasion of many cancer cells [11]. Among the MMPs, increased activity of the gelatinases MMP-2 and MMP-9 was found in colon tissue samples obtained from CRC patients [12]. In vitro studies have suggested that platelets can induce p38MAPK phosphorylation and MMP-9 upregulation in colorectal cell lines, with subsequent cell-invasion-promoting effects [13]. It remains unknown whether PMPs, as the product of platelet activation, can affect CRC cells in the manner described above.

In the present study, we investigated the impact of platelet-derived microparticles on the CRC cell levels of the chemokine receptor CXCR4 as well as surface integrins, including platelet-specific receptors, and investigated how incorporated PMPs affect the migratory and invasive potential of CRC cells. Finally, we evaluated whether PMPs can stimulate colorectal cancer cell invasiveness by enhancing MMP-2/MMP-9 levels and activity via the p38MAPK pathway.

## Methods

### Cell culture

Human colorectal adenocarcinoma cell lines with different phenotype and migratory potentials, HT29 (epithelial-like), SW480 (mesenchymal-like) and SW620 (strongly mesenchymal-like), were purchased from the American Type Culture Collection (ATCC, Manassas, VA, USA) and cultured in appropriate medium with 10% FBS and antibiotics (Additional file 2: Supplementary Methods) at the constant conditions of 37 °C, 5% CO_2_ and 95% humidity.

### Isolation of PMPs

PMPs were obtained from outdated concentrates of human blood platelets obtained from the Regional Centre of Blood Donation and Blood Treatment in Łódź in accordance with applicable law. PMPs were obtained from platelets after several steps of centrifugation and ultracentrifugation (Additional file 2: Supplementary Methods). To provide donor viability, outdated concentrates of human blood platelets were collected from donors with different blood groups.

### Confocal imaging

PMPs were labelled using PKH67 Fluorescent Cell Linker kit (Sigma Aldrich, St. Louis, MO, USA) according to the manufacturer’s instructions and incubated with CRC cells (50 µg of PMPs per 10^6^ cells and at a final concentration of 100 µg/ml PMPs) for 4 h. The cell components were labelled after the addition of Alexa Fluor 594-conjugated wheat germ agglutinin (5 µg/ml, plasma membrane labelling dye) and Hoechst 33,342 (5 µg/ml, cell-permeant nuclear dye). In another set of experiments, after incubation with PMPs, cells were labelled with PKH67, and antibodies against CXCR4 (10 µg per 10^6^ cells) or CD61 (10 µg per 10^6^ cells) were added followed by incubation with Hoechst 33,342 (5 µg/ml) (Additional file 2: Supplementary Methods).

### Wound healing, migration and invasion assays

CRC cells were seeded in 24-well plates and grown in culture medium. At 90% confluence, the medium was replaced with medium without FBS, containing or not containing (control) PMPs, and the cells were incubated for 4 h at 37 °C in a humidified atmosphere with 5% CO_2_. To determine whether migration was CXCR4-dependent, cells were incubated with the CXCR4 antagonist AMD3100 (at a final concentration of 10 μM) or with anti-CXCR4 antibodies (at a final concentration of 100 μg/ml). SDF-1 (at a final concentration of 400 ng/ml) was added after incubation of CRC cells with PMPS to stimulate CXCR4-dependent cell migration. Cell migration was evaluated by measuring the cell-free surface at the beginning of the experiment (immediately after the scratch was made) and every 2 h until a 24-h period (Additional file 2: Supplementary Methods). The migration and invasion properties of tumour cells labelled with CellTracker after incorporation of PMPs were evaluated in uncoated or Matrigel-coated (10 mg/ml, Thermo Fisher Scientific) Boyden chambers, respectively (Additional file 2: Supplementary Methods).

### Proliferation assay

CRC cells were seeded in 96-well plates, grown in culture medium and incubated with or without (control) PMPs in appropriate medium not supplemented with FBS for 4 h at 37 °C in a humidified atmosphere with 5% CO2. Proliferation was measured on the basis of DNA content without relying on metabolic activity using a CyQuant Proliferation Assay Kit (Thermo Fisher Scientific, Waltham, MA, USA) according to the manufacturer’s instructions.

### Immunoblotting

CRC cells were incubated for 4 h with PMPs as described above for the detection of CXCR4, MMP-2, and MMP-9 and lysed using RIPA buffer (Sigma Aldrich) or incubated for 10 min with PMPs for the detection of nonphosphorylated and phosphorylated ERK1/2 and p38MAPK and lysed with M-PER Mammalian Protein Extraction Reagent (Thermo Fisher Scientific). The lysates (25 µg of protein) were then separated on an SDS‒PAGE 10% polyacrylamide gel, transferred to a nitrocellulose membrane and incubated with rabbit anti-human antibodies against nonphosphorylated p38MAPK (Cell Signaling) and ERK1/2 (Thermo Fisher Scientific) and phosphorylated p38MAPK (Cell Signaling) or mouse anti-human antibodies against CXCR4 (Thermo Fisher Scientific), MMP-2, MMP-9 (Thermo Fisher Scientific) and phosphorylated ERK1/2 (Thermo Fisher Scientific), followed by incubation with secondary goat anti-mouse or goat-anti-rabbit IgG (Santa Cruz Biotechnology) conjugated with horseradish peroxidase (HRP) (Additional file 2: Supplementary Methods).

### Flow cytometry

CRC cells were seeded in 24-well plates and grown in culture medium. At 90% confluence, the medium was replaced with medium without FBS, containing or not containing (control) PMPs. The cells were incubated for 4 h at 37 °C in a humidified atmosphere with 5% CO_2_ and detached from the plate using Accutase (Sigma Aldrich). A total of 10^6^ cells per millilitre were incubated with 10 µg of anti-CXCR4 PE-conjugated antibodies (clone 12G5, Thermo Fisher Scientific) or FITC- or PE-conjugated antibodies against the integrin subunits α2, α6, αv, β3, β4, αIIβ or glycoprotein Ib and fixed with 1% Cellfix (BD Biosciences) for surface detection or permeabilized by the addition of 1% Tween 20 for intracellular detection (Additional file 2: Supplementary Methods).

### MMP2 and MMP-9 activity assays

CRC cells were incubated with PMPs as described above, and 50 × 10^3^ cells per ml were added to FITC-labelled gelatine-coated wells (0.1 mg/ml) and incubated for 24 h. The gelatinolytic activity of MMP-2 and MMP-9 was measured as green fluorescence derived from degraded gelatine (Additional file 2: Supplementary Methods). In another set of experiments, MMP-2 and MMP-9 activity was measured as CRC cell migration through 0.2% gelatin-coated filters in the Boyden chambers as described above (Additional file 2: Supplementary Methods). The gelatinolytic properties of CRC cell were blocked by the appropriate inhibitors of MMP-2 (ARP101) or MMP-9 (CTK8G1150) at a final concentration of 20 µM or by the specific inhibitor of p38MAPK (SB202190, Cell Signaling Technology) at a final concentration of 5 µM.

### Enzyme-linked immunosorbent assay for MMP-9

HT29, SW480 and SW630 cells were incubated with PMPs as described above, and the MMP-9 concentration was evaluated in the undiluted conditioned medium using ELISA according to the manufacturer’s instructions (Biotechne) (Additional file 2: Supplementary Methods).

### Statistical analysis

Data are presented as the mean, standard error and min–max values or as median and the interquartile range (IRQ, lower [25%] quartile to upper [75%] quartile, depending on the data scale and distribution. The normality of the data distributions was verified with the Shapiro‒Wilk test; variance homogeneity was tested with Levene’s test, and the significance of differences was evaluated using the Mann‒Whitney U test or Student’s t test for independent samples, depending on normality (Additional file 2: Supplementary Methods).

## Results

### PMPs incorporate into CRC cells and transport platelet-specific receptors

**Fig. 1:**
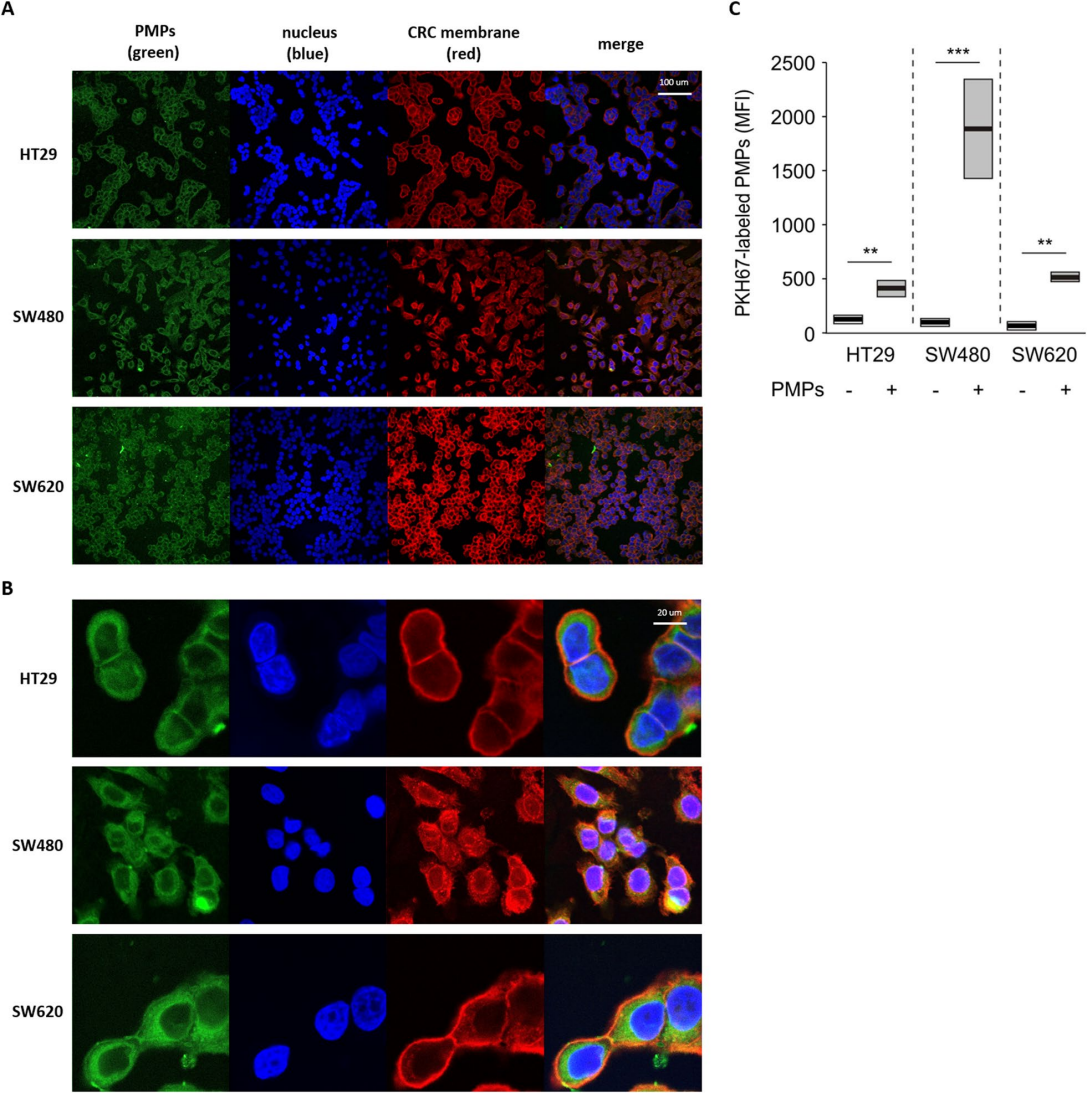
The internalization of platelet-derived microparticles by colorectal cancer cells. **A** 20× and **B** 100× magnification: representative confocal microscopy images of PKH67-labelled PMPs uptake (green) by HT29, SW480 and SW620 cells during 4 h of incubation. Staining with Hoechst 33,342 (blue) for nuclei and Alexa Fluor 594 (red) wheat germ agglutinin for cell membrane C – flow cytometry analysis of PKH67-labelled PMP internalization. The results are presented as the mean (vertical line) and standard error (box). Significance estimated with the nonparametric U-Man-Whitney test: ***P < 0.001, **P < 0.01, N = 5

**Fig. 2:**
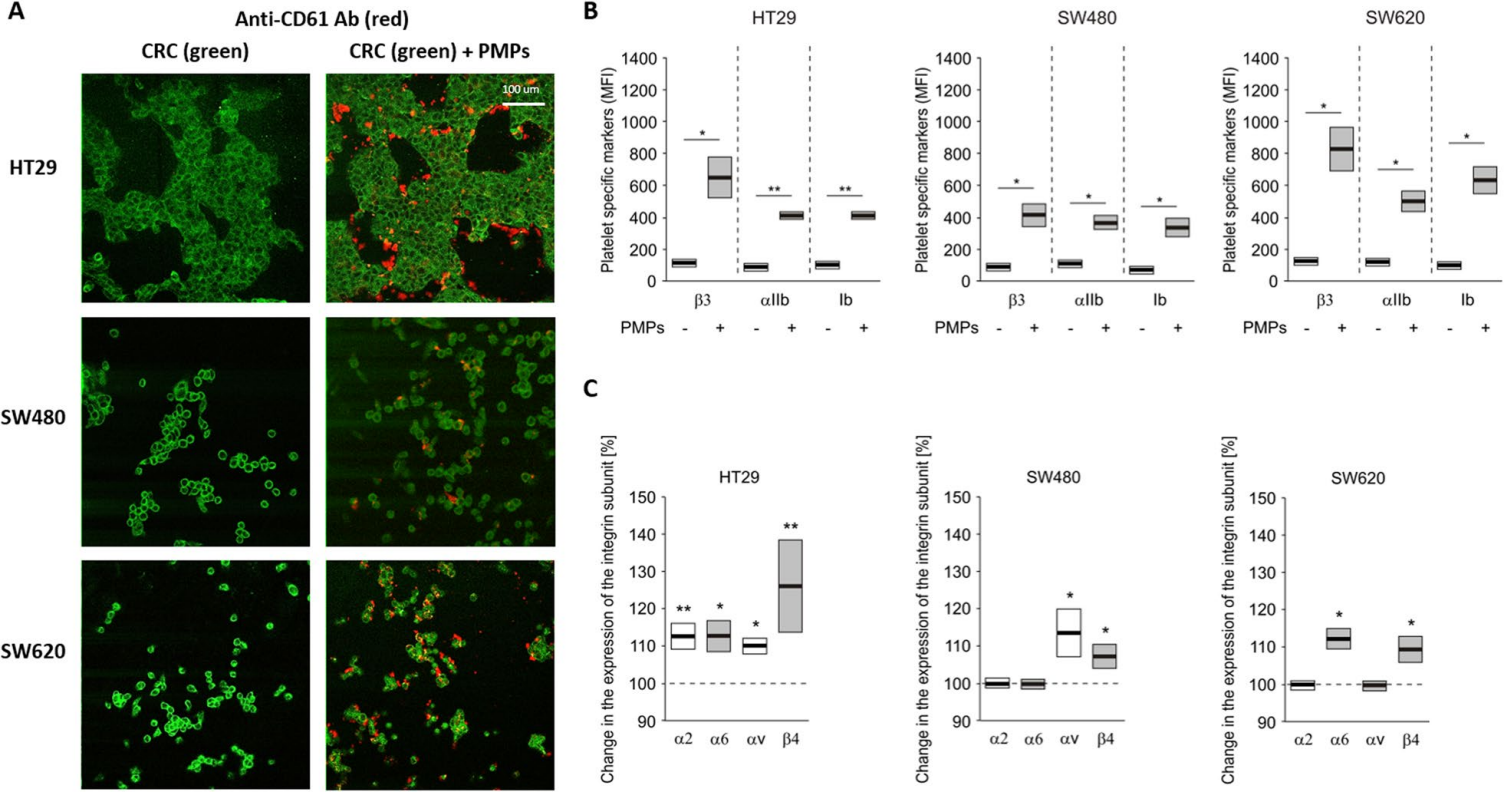
PMPs transfer platelet-specific antigens and stimulate the expression of integrin subunits. **A** Representative confocal microscopy images of β3 (anti-CD61 Ab, red) detection in HT29, SW480 and SW620 cells not treated (left) or incubated (right) with PMPs labelled with PKH67 (green) dye. **B**, **C** flow cytometry analysis (presented as in Fig. 1) of integrin subunits (β3, αIIb, α2, α6, αv, and β4) and glycoprotein Ib on the surface of CRC cells incubated with PMPs compared to control cells (100%—dash horizontal bar). Significance was estimated as presented in Fig. 1, **P < 0.01, *P < 0.05, N = 5

Many tumours, including CRC, contain heterogeneous mixture of cells of dissimilar characteristics. Cancer cells also differ among patients and even within the same patient [14]. The existence of heterogeneous populations of cancer cells means that there are different growth rates and invasive potentials [15]. Thus, to study the mechanisms of CRC development and metastasis, it appears reasonable to use more than one colorectal cancer cell line with different phenotypes. The HT29, SW480 and SW620 cell lines used in our research are commonly studied types of CRC cells. HT29 exhibits epithelial characteristics, while the mesenchymal-like cell lines SW480 and SW620 originate from the same patient and are derived either from the primary tumour (SW480) and from metastatic lesions in lymph nodes (SW620) [16]. We examined the incorporation of PMPs into CRC cells by confocal imaging and flow cytometry. Confocal analysis revealed that PMPs, following 4 h incubation, were internalised into HT29, SW480 and SW620 cells (Fig. 1A, B). PMPs labelled with PKH67 were incubated with CRC cells for various times (30 min, 1 h, 2 h, 4 h), and as shown by flow cytometry, the efficiency of PMP incorporation was similar for HT29 and SW620 cells but slightly higher for SW480 cells (Fig. 1C). We found that the uptake of PMPs increased over time (Additional file 1: Fig. 1S). Furthermore, PMPs transferred the specific platelet integrin subunits or glycoproteins to the surface of CRC cells; the presence of αIIb, β3 integrin subunits and glycoprotein Ib was observed on CRC cells only after the uptake of PMPs (Fig. 2A, B), and they were not detected in control CRC cells.

Moreover, the incorporation of PMP also increased the surface level of some integrin subunits that are normally present on the colorectal cell outer membrane, and this effect was, to some extent, cell type-dependent (Fig. 2C). Elevated presence of integrin subunits α2, α6, αv, and β4 was observed in HT29 cells, but not all these integrin subunits were increased in SW480 or SW620 cells after PMP uptake (Fig. 2C).

### The effect of PMPs on the surface and intracellular levels of CXCR4 and on CXCR4-dependent migration

**Fig. 3:**
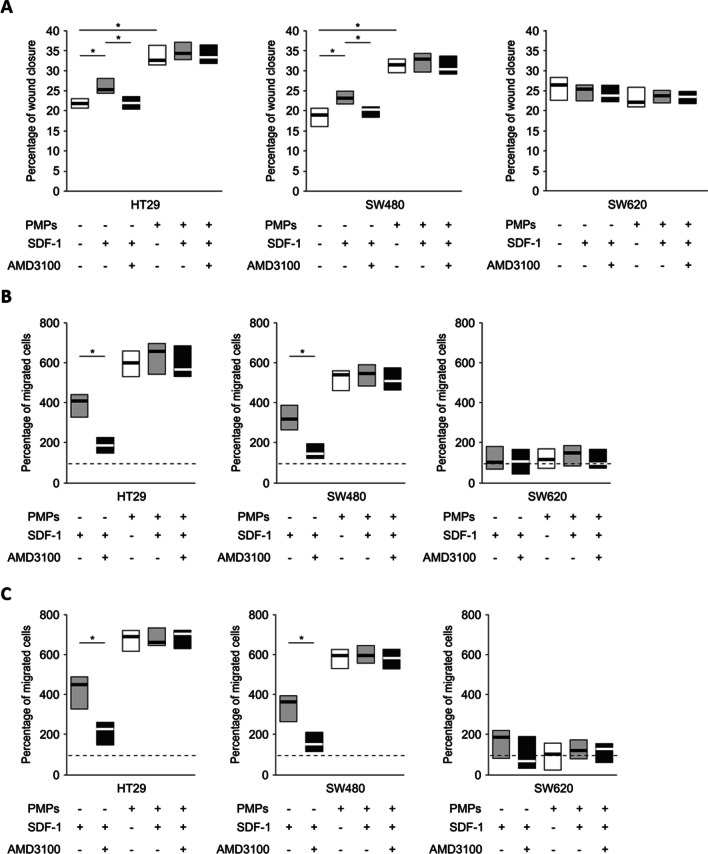
Effect of PMP internalization on the migration and invasion potential of HT29, SW480 and SW620 cells. **A** Wound closure (recovery) of CRC cells, control and preincubated for 4 h with PMPs after a 24-h migration period. *P < 0.05, N = 5. **B** and **C** migration and invasion, respectively, of HT29, SW480 and SW620 cells through uncoated (**B**—migration) or Matrigel-coated (**C**—invasion) after 4 h of incubation with PMPs in the presence or absence of SDF-1 and/or the CXCR4 antagonist AMD3100. For **B** and **C**, nontreated cells were considered 100% (dashed horizontal bar). The asterisk denotes the significance of the inhibition of migration and invasion of cells treated with AMD3100. Statistics were calculated with one-way ANOVA followed by Tukey’s test for multiple comparisons: *P < 0.05, N = 5

Flow cytometric identification of surface CXCR4 indicated much lower level of this receptor on SW620 than on SW480 and HT29 cells, and addition of PMPs did not change the surface presence of CXCR4 for either cell line (Additional file 1: Fig. 2SA). Moreover, there was no significant difference in the intracellular level of CXCR4 between HT29, SW480 and SW620 cells, regardless of whether PMPs were incorporated into those cell lines (Additional file 1: Fig. 2SB). Immunoblots of the whole cell lysates confirmed the flow cytometric results; there was no significant effect of PMPs on the level of CXCR4 (Additional file 1: Fig. 2SC). Additionally, the intensity of the bands corresponding to various CXCR4 forms differed between HT29 cells (epithelial-like) and SW620 cells (mesenchymal-like) (Additional file 1: Fig. 2SC). In the wound-healing assay, the migration of HT29 and SW480 cells was increased after the incorporation of PMPs (Fig. 3A). In contrast to HT29 and SW480 cells, we observed no change in the migration of SW620 cells following incubation with PMPs (Fig. 3A). (representative images of wound healing assay are presented in Additional file 1: Fig. 3S). We also found that PMPs stimulated the motility and invasiveness of HT29 and SW480 cells to a greater extent than after activation of CXCR4 by its ligand, SDF-1/CXCL12 (Fig. 3B, C). In the presence of the CXCR4 antagonist AMD3100, cell migration and invasion across Matrigel were significantly reduced for HT29 and SW480 cells stimulated with SDF-1. However, there was no inhibitory effect of AMD3100 on HT29 and SW480 cells containing PMPs and stimulated with SDF-1. Activation or inhibition of CXCR4 by its ligand SDF-1 or AMD3100, respectively, had no effect on the migration and invasion rates of SW620 cells (Fig. 3B, C) (representative images of cell migration assay through un-coated and Matrigel-coated Boyden chambers are presented in Additional file 1: Figs. 4S and 5S).

### The effect of PMPs on CRC cell proliferation

Our results showed an increasing proliferation rate of HT29 and SW620 cells after the tested periods of time (24, 48, 72, 96 h). We did not reveal any significant effect of PMPs on this feature for either cell line, but the tendency suggesting reduced proliferation after PMPs incorporation was observed for SW620 cells (Additional file 1: Fig. 6S).

### The effect of PMPs on the activity of MMP-2 and MMP-9

**Fig. 4:**
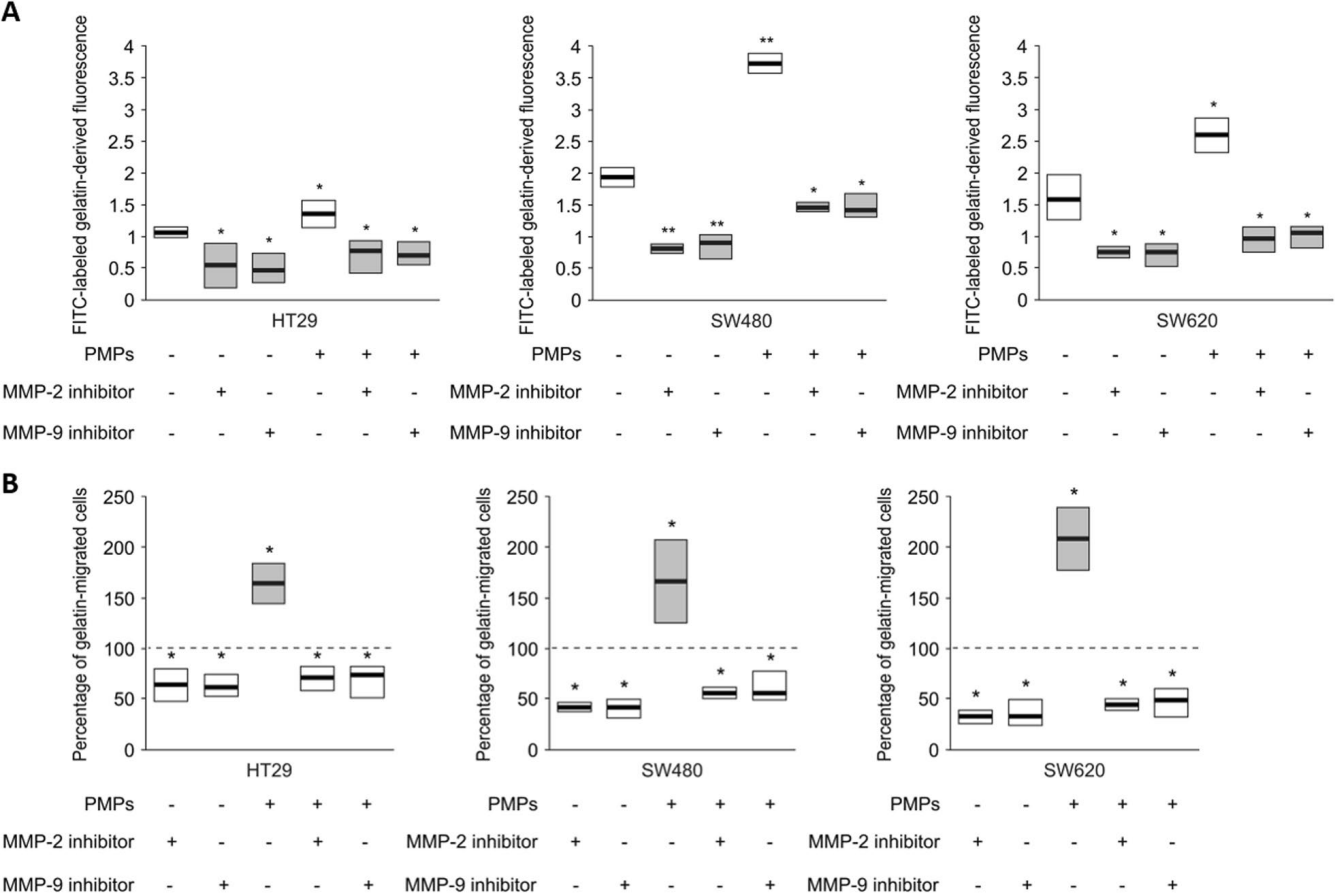
Activity of metalloproteinases in CRC cells after PMP uptake. **A** Degradation of FITC-labelled gelatine by CRC cells. **B** Migration of CRC cells through gelatine-coated Boyden chambers in the absence or presence of MMP-2 or MMP-9 inhibitors (ARP101 or CTK8G1150, respectively). Quantified data (presented and statistically analysed as in Fig. 1) of the percentage of gelatine-migrated cells compared to the control (nontreated cells, 100%, marked with dashed horizontal bars.). **P < 0.01, *P < 0.05, N = 5

Next, we studied the effect of PMPs on the activity of MMPs in HT29, SW480 and SW620 cells. We observed increased degradation of fluorescent-labelled gelatine (Fig. 4A) as well as increased migration of CRC cells through membranes covered with gelatine (Fig. 4B) after PMP uptake compared to nontreated cells.

The effect of PMPs on the gelatinolytic properties was observed for all tested cell lines, but the highest was noted for SW480 and SW620 cells. Inhibitors of MMP-2 or MMP-9 almost completely reduced PMP-induced gelatine degradation by CRC cells and their migration through gelatine (Fig. 4A, B) (representative images of cell invassion assay through gelatin-coated Boyden chambers are presented in Additional file 1: Fig. 7S).

### The effect of PMPs on cell signalling

**Fig. 5:**
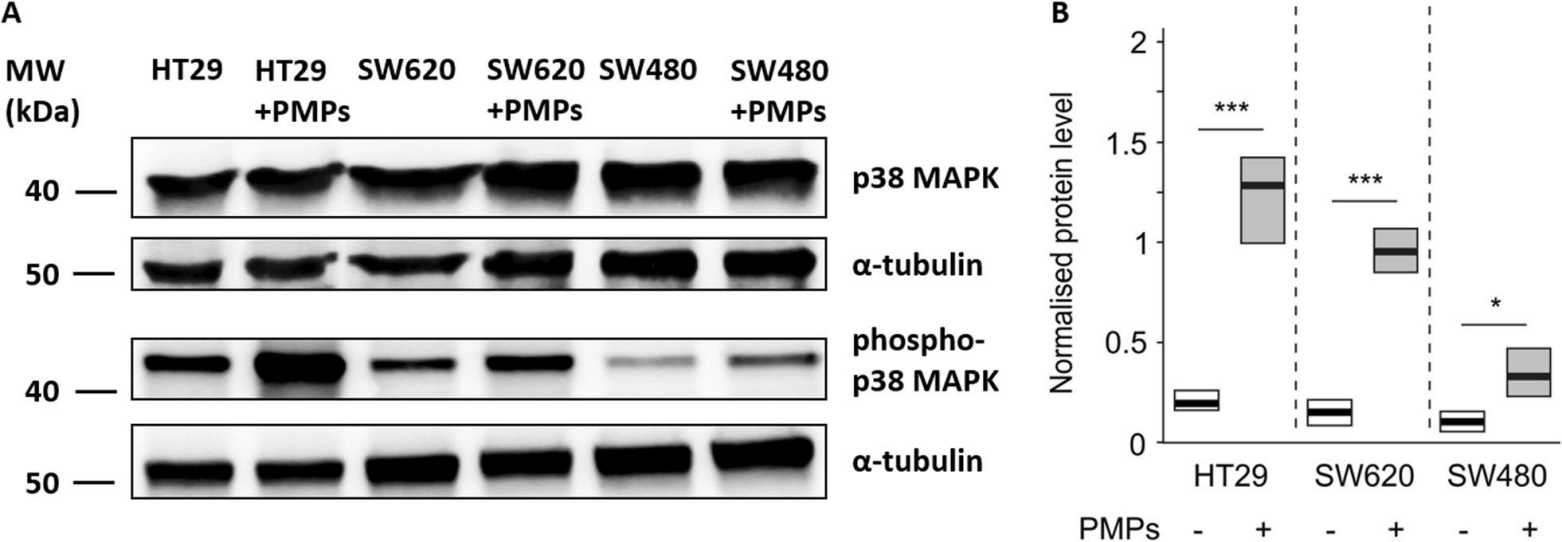
Effect of PMPs on p38MAPK phosphorylation. **A** Representative Western blots of phosphorylated p38MAPK (phospho-p38) and total p38MAPK (p38) in CRC cells before and after 10 min of incubation with PMPs. **B** Normalized densitometric values of phospho-p38 and the total p38MAPK ratio. Quantified data are presented and statistically analysed as in Fig. 1. ***P < 0.001, *P < 0.05, N = 5

We observed elevated phosphorylation of p38MAPK in HT29, SW480 and SW620 cells after incubation with PMPs compared to nontreated cells (Fig. 5A, B). Interestingly, the highest PMP-induced phosphorylation of this kinase was observed for HT29 cells, while the lowest was observed for SW480 cells. In contrast, we did not obtain significant changes in the phosphorylation of ERK1/2 proteins in any CRC cell line after PMP uptake (Additional file 1: Fig. 8S).

### PMP-induced CRC invasiveness is dependent on the p38MAPK pathway

**Fig. 6:**
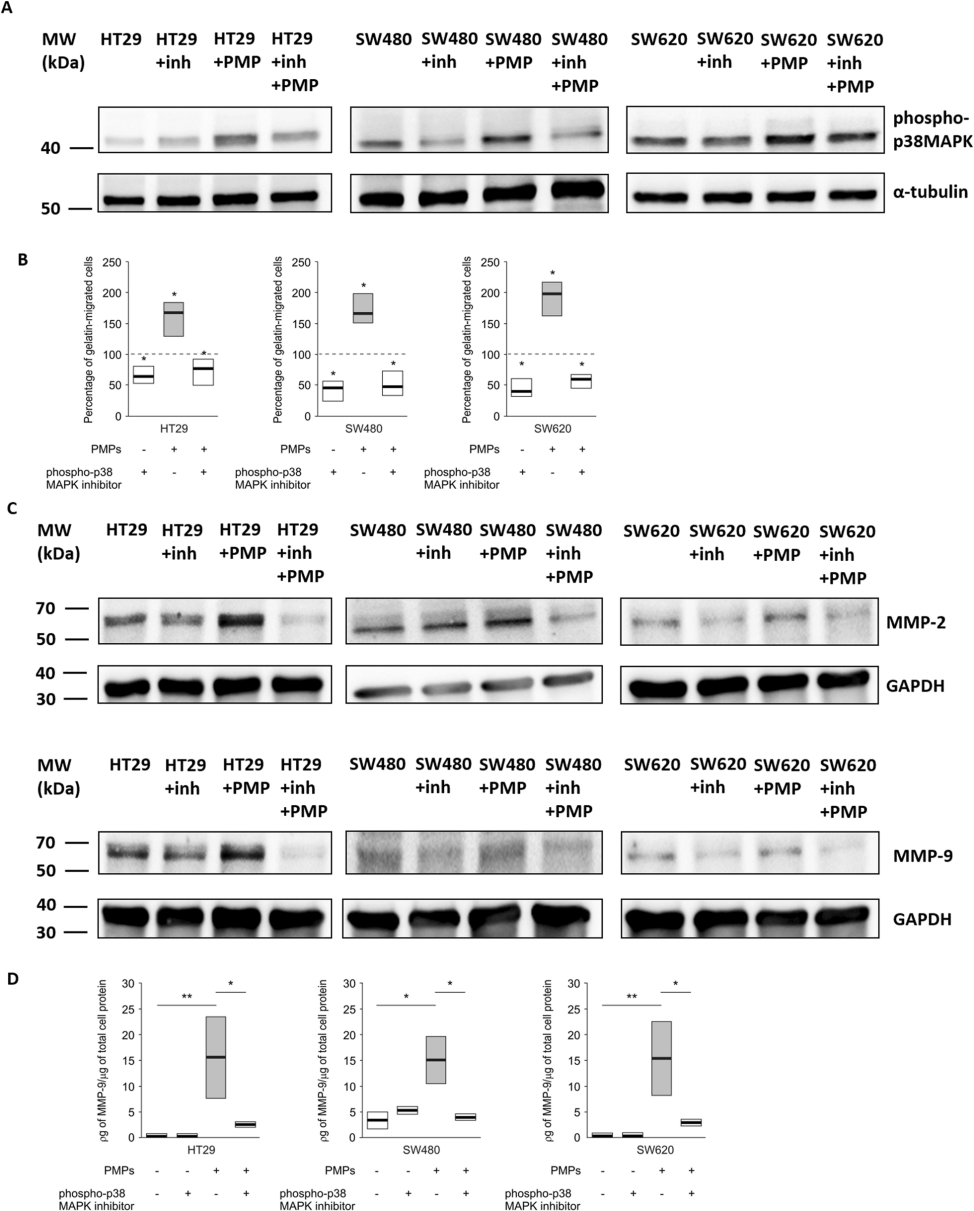
Inhibition of p38MAPK phosphorylation and its effect on CRC cell migration and MMP-2/MMP-9 level/release after PMP uptake. **A** Representative Western blots of phospho-p38MAPK in CRC cells after 10 min of incubation with PMPs in the absence or presence of the inhibitor SB202190. **B** Migration of CRC cells through gelatine-coated Boyden chambers after 4 h of incubation with the inhibitor SB202190. Quantified data (presented and statistically analysed as in Fig. 1) of the percentage of gelatine-migrated cells compared to the control (nontreated cells, 100%, marked with dashed horizontal bars). *P < 0.05, N = 5. **C** Representative Western blots of MMP-2 and MMP-9 in CRC cells after 4 h of incubation with the inhibitor SB202190. **D** Amount of MMP-9 released from CRC into conditioned medium after 4 h of incubation with PMPs in the absence or presence of the inhibitor SB202190, as measured by ELISA. The data are presented and statistically analysed as in Fig. 1. **P < 0.01, *P < 0.05. N = 6

Because we observed that PMP increased both the level of MMP-2/-9 proteins and the phosphorylation of p38MAPK, we investigated the effect of SB202190, an inhibitor of the p38MAPK pathway, on the invasive potential of CRC cells after PMP uptake. As shown in Fig. 6A, SB202190 reduced PMP-induced phosphorylation of p38MAPK in all cell lines studied. The elevated migration of HT29, SW480 and SW620 cells after PMP uptake through membranes coated with gelatine was inhibited by SB202190 (Fig. 6B). Figure 6C shows that the incorporation of PMPs increased the protein levels of MMP-2 and MMP-9 in all three CRC cell lines. We found that PMPs do not contain MMP-9 although resting and thrombin stimulated platelets produce MMP-9 (Additional file 1: Fig. 9S). Moreover, SB202190 reduced PMP-stimulated level of MMP-2 and MMP-9 in those cells (Fig. 6C) (representative images of cell invassion assay through gelatin-coated Boyden chambers are presented in Additional file 1: Fig. 10S). Furthermore, we found the increased amount of MMP-9 in the conditioned medium of HT29, SW480 and SW620 cells after their incubation with PMPs, and this effect was reduced when SB202190 was added together with PMPs (Fig. 6D). In contrast, PMPs had no effect on the protein level of MMP-14, which is important for the activation of MMP-2 (Additional file 1: Fig. 11S).

## Discussion

We have demonstrated here that PMPs can be incorporated into CRC cells that are either in epithelial- or mesenchymal-like and can affect the migration and invasion of those cells. The incorporation was time dependent; short-lasting incubation up to 30 min was not sufficient for the uptake of PMPs, while 4 h of incubation showed substantial incorporation and the increase in the invasive properties and mobility of CRC cells. Additionally, different amounts of PMPs were incorporated by various CRC cell lines. Experimental evidence suggests that PMPs, like most extracellular vesicles, are usually taken up into endosomal compartments via endocytosis, mainly caveolin-dependent endocytosis (CDE) [17]. Caveolin is present at different levels on the surface of some commonly studied CRC cell lines. [18]. Higher caveolin level was shown for mesenchymal-like cells compared to epithelial-like cells [19, 20]. This finding may explain our observation that SW480 cells incorporate PMPs more efficiently than epithelial-like HT29 cells. However, because the incorporation of PMPs does not occur through a single mechanism and is a complicated process, other pathways of microvesicles uptake cannot be excluded. Blocking the particular mechanism of PMP uptake by specific inhibitors should shed more light on the dominant mechanism of PMP incorporation into CRC and will be performed in future studies.

Analysis of selected platelet integrin subunits showed that incorporated PMPs transfer these receptors to CRC cells, especially integrins that support the formation of metastases (β3 integrin subunit) [21]. Additionally, incorporation of PMPs led to the increased surface presence of several integrin subunits that are normally present on colorectal cancer cells, such as α2 or αv, which are known to be involved in the metastatic process ([22], and [23], respectively). Because these integrin subunits are not present in platelets, they cannot be simply transferred by PMPs into cells. Increased level of these subunits probably results from de novo protein synthesis or elevated exposure of these subunits on the cell surface.

We observed higher amount of CXCR4, a receptor for SDF-1, on epithelial-like (HT29) and mesenchymal-like (SW480) cell surfaces than on strongly mesenchymal-like SW620 cells. It has been previously reported that CXCR4 level is elevated in various cancer cell types [24, 25]. As it was shown by RT‒PCR, CXCR4 is found mostly in HT29 cells, with low expression in SW620 cells [26]. The metastasis of certain cancers has been postulated to be directly regulated by SDF-1 because high levels of SDF-1 are also found in metastatic sites [27]. Indeed, we observed that the migration and invasion of epithelial-like HT29 and mesenchymal-like SW480 cells were inhibited by a CXCR4 inhibitor. Incorporation of PMPs affected neither the surface nor intracellular level of CXCR4 in HT29, SW480 and SW620 cells. Furthermore, the increased migration and invasion rates of HT29 and SW480 cells after the incorporation of PMPs were not dependent on the SDF1/CXCR4 pathway. We concluded that, although PMPs can modulate phenotype of CRC cells to become more migrative/invasive and have less effect on strongly mesenchymal-like cancer cells, it is not linked to the ability of PMPs to transfer CXCR4 into cells and cannot be attributed to the increased effectiveness of the CXCR4/SDF1 axis. Our findings indicate that the effect of PMPs is in some way cell type-dependent even between cells from the same cancer but with different epithelial-like or mesenchymal-like phenotypes. These results appear to be in contrast to other reports about normal and malignant haematopoietic and breast cancer cells, showing that PMPs can transfer CXCR4 to the cell surface and this increase level of CXCR4 correlates with enhancement of the chemotactic responses of those cancer cells to SDF-1 [28]. The molecular mechanism of the PMP-related increase in the responsiveness of cancer cells to SDF-1 has not yet been elucidated. It was suggested that this finding could be the result of a priming effect (involving CXCR4 from the cancer cells through an increase in the incorporation of CXCR4 into membrane lipid rafts), as was reported for other molecules [29], or the result of a transfer of functional CXCR4 from the platelets to the recipient cancer cells. Our data, however, confirm that inhibition of the CXCR4/SDF-1 axis with the CXCR4 antagonist AMD3100 could become a therapy for preventing CRC metastasis. It was recently suggested that knockdown of CXCR4 (with RNA interference or pharmacological inhibition with AMD3100) substantially limited orthotopic growth of breast cancer cells in vivo and prevented the development of macroscopically detectable metastases [30]. Such a therapy can therefore be effective in colorectal cancer as well, at least in its early stages.

As presented previously, the interaction of PMPs with normal and malignant human haematopoietic cells, as well as with lung and breast cancer cell lines, resulted in the activation of MAPK p42/44 and AKT, thus indicating that PMPs are able to interact directly with target cells and act as signalling molecules [8, 28]. Zara and colleagues [31] observed increased phosphorylation of p38MAPK but no activation of other intracellular signalling pathways, such as ERK or Akt, after incorporation of PMPs in the highly aggressive breast cancer cell line MDA-MB-231 compared to the less aggressive MCF-7-cell line. The above examples show that the effect on PMPs as signal molecules in intracellular pathways can be different in various cancer cells, depending not only on the phenotype of the cells but also on the cancer origin. p38MAPK is implicated in the control of cell motility; thus, increased phosphorylation of this protein induced by PMPs can lead to expanded migration. After incorporation, PMPs can affect recipient cells by at least two mechanisms. One is dependent on the proteins that are transferred by PMPs, especially the surface receptors of recipient cells, and increases the total amount of particular surface receptors and thus enhances the cellular response to ligand‒receptor interactions. In the other mechanism, PMPs serve as signalling molecules that activate intracellular pathways leading to increased level and activity of different proteins, including MMPs, which we have also shown here. Increased levels of MMPs have been found in various types of cancer, and a strong positive correlation has been shown between MMPs and cancer progression and metastasis. Moreover, it has also been shown that PMPs can stimulate MMP-2 activation and secretion, MMP-14 expression or MMP-dependent cell migration in lung [8], breast [32] and prostate cancer [33]. In these studies, we revealed increased levels and activity of MMP-2 and MMP-9 in CRC cells after the incorporation of PMPs. Moreover, we also found elevated amounts of MMP-9 that had been released by CRC cells into conditioned medium during incubation with PMPs. This finding suggests that PMPs not only stimulate the expression of MMP-2/-9 proteins but also induce the release of activated forms of those metalloproteases that are able to degrade the extracellular matrix. The fact that PMPs increase the protein level of MMP-9 seems even more interesting when considering that gene transcription of MMP-9 is inducible and the promoter region is highly responsive to growth factors and cytokines in several cells [34]. There are no evidences in the literature showing that PMPs contain MMP-2 or MMP-9, although MMP-9 can be produced by platelets, especialy after their stimulation. We did not found MMP-9 in the PMPs but we observed presence of this protein in the resting and thrombin stimulated platelets. This indicate that PMPs stimulate expression of MMP-9 rather than transfer this protein into recipient CRC cells.

To explain the mechanism by which PMPs can stimulate MMP-2/MMP-9 expression in CRC cells, we studied the possible role of p38MAPK pathway activation. Our results show that PMPs can induce the phosphorylation of p38MAPK in CRC cells regardless of their phenotype. Moreover, inhibition of p38MAPK phosphorylation effectively reduced MMP-2 and MMP-9 level and, in parallel, MMP-dependent migration of CRC cells. Additionally, the PMP-induced elevation of MMP-9 released from CRC cells was abrogated when an inhibitor of p38MAPK phosphorylation was added. Thus, our results indicate that PMPs can stimulate CRC cell invasive potential by inducing MMP-2 and/or MMP-9 expression and activity via phosphorylation of p38MAPK protein. To date, similar observations have been made for CRC and platelets only [13]. Interaction with platelets can increase the level of MMP-9 as well as the phosphorylation of p38MAPK in HT29 and CaCo-2 cells, but we show here that PMPs can also stimulate the same effect in mesenchymal-like CRC cells. Our findings are in agreement with other studies, which showed that p38MAPK inhibition resulted in decreased MMP-9 activity in leukaemia and lung cancer cells [35, 36]. It has been shown that the observed effect of platelets on the p38MAPK-MMP-9 axis is related to thrombospondin 1 (TSP1) and clusterin activity [13]. Platelet-secreted TSP1 and clusterin promote the signal regulation of MMP-9 in platelet-induced CRC cell invasion via a p38MAPK-regulated pathway. Because both TSP1 and clusterin are also present in PMPs, similar mechanisms for the effects of platelets and PMPs on the p38MAPK-MMP-9 pathway cannot be excluded and should be further studied.

## Conclusions

In conclusion, we show that platelet-derived microparticles can be incorporated by CRC cells, transferring not only integrins but also stimulating the expression of integrin subunits already existing on the CRC cell surface to increase mobility and invasive properties of epithelial-like and also mesenchymal-like CRC cells. Moreover, we revealed that PMPs can stimulate MMP-2 and MMP-9 levels and activity, leading to increased CRC cell invasiveness through p38MAPK pathway activation. These data provide evidence of the therapeutic potential of modulating not only platelet-CRC but also PMP-CRC cell interactions by focusing on p38MAPK signalling and may be a new viable approach to preventing and reducing metastasis in CRC.

## Supplementary Information

**Additional file 1:** Supplementary Results.

**Additional file 2:** Supplementary Methods.

## Data Availability

The data generated in this study are presented within the article and its supplementary data files. All microscopy slides, raw western blot data and data generated in this study are available upon request. All data generated or analysed during this study are included in this published article**.**

## Abbreviations

CRC: Colorectal cancer
PMPs: Platelet-derived microparticles
MMPs: Metalloproteases
ECM: Extracellular matrix
p38MAPK: Mitogen-activated protein kinase p38
CXCR4: C-X-C chemokine receptor type 4
SDF1: Stromal cell-derived factor 1
