# Supplementary material for: Platelet-derived microparticles stimulate the invasiveness of colorectal cancer cells via the p38MAPK-MMP-2/MMP-9 axis
Source: Cell Commun Signal. 2023 Mar 7;21:51. doi: 10.1186/s12964-023-01066-8 (PMC9990213; doi:10.1186/s12964-023-01066-8)
Supplement: Supplementary file 2 — Additional file 1: Supplementary Results. [file 12964_2023_1066_MOESM2_ESM.docx]

**Supplementary Figures**

**
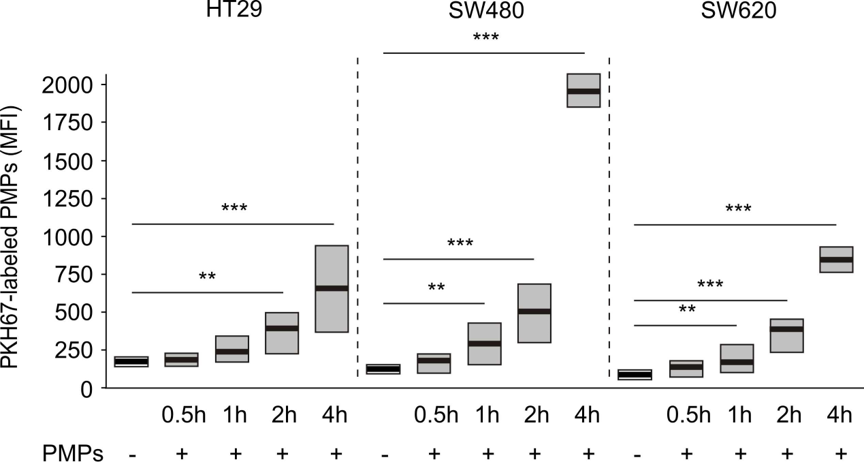
**

**Fig. 1S. The internalization of platelet-derived microparticles by colorectal cancer cell lines.** Flow cytometry analysis of PKH67-labelled PMPs internalization into HT29, SW480 and SW620 cells during the 0.5-h, 1-h, 2-h and 4-h incubation periods. Quantified data (presented and statistically analysed as in Fig. 1) of the PKH67 mean fluorescence intensity. ** P<0.01, *** P < 0.001, N = 3.

**
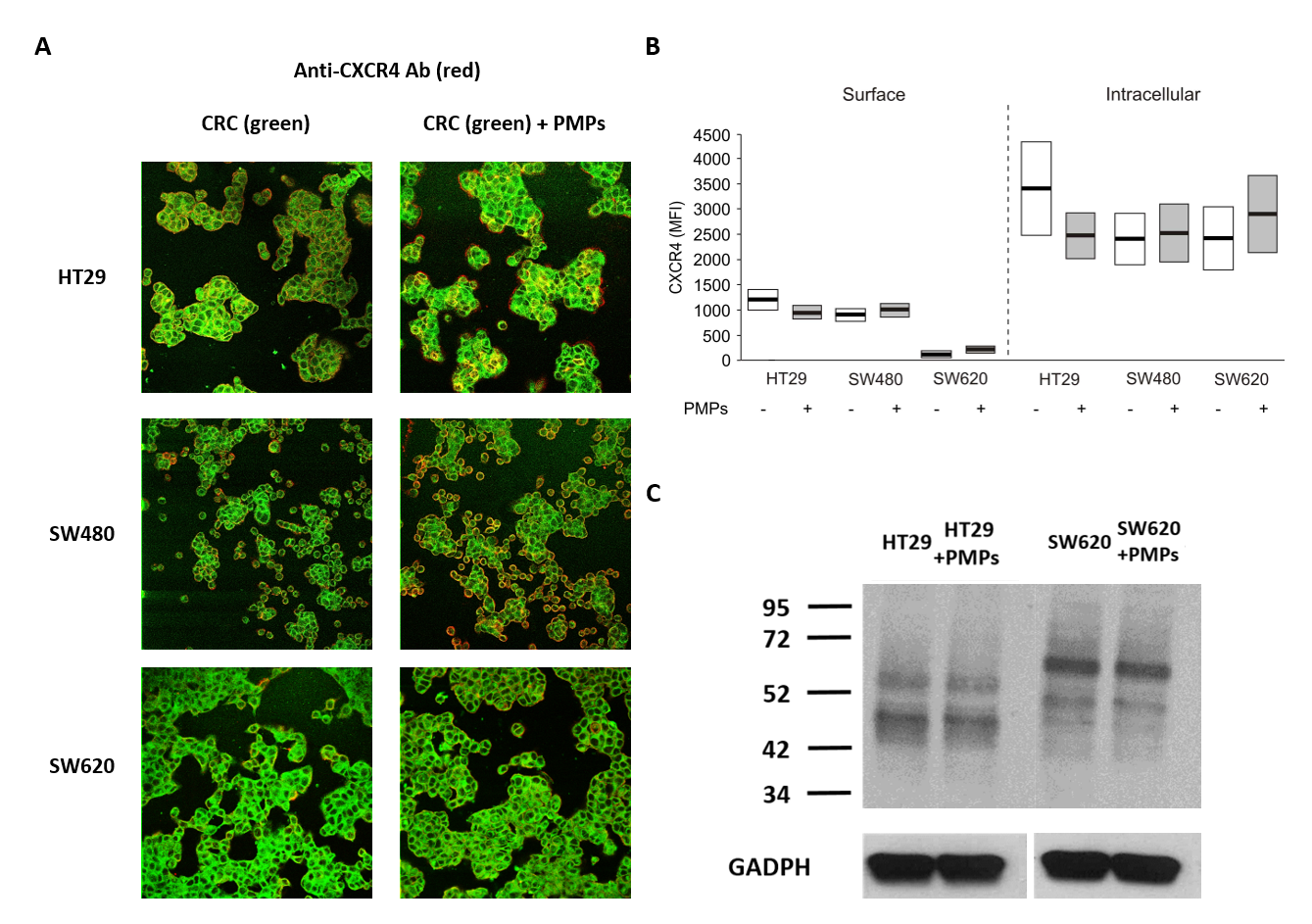
**

**Fig. 2S. Surface and intracellular CXCR4 expression on CRC cells after PMP uptake.** A - Representative confocal microscopy images of CXCR4 detection in HT29, SW480 and SW620 cells that were not incubated (left images) or were incubated (right images) with PMPs (at a dose of 50 µg of PMPs per 10^6^ cells and at a final concentration of 100 µg/ml) in appropriate medium not supplemented with FBS for 4 h at 37 °C in a humidified atmosphere with 5% CO_2_. CXCR4 was identified with monoclonal antibodies conjugated with phycoerythrin (10 µg of Ab per 10^6^ cells), while cell membranes were labelled with PKH67 dye. B - Flow cytometry analysis of CXCR4 expression on the surface of CRC cells incubated with PMPs. Quantified data (presented and statistically analysed as in Fig. 1) of the mean fluorescence intensity of antibodies bound to CXCR4, N = 5. C – Representative western blots of CXCR4 expression in cell lysates of HT29 and SW620 cells after PMP uptake.

**
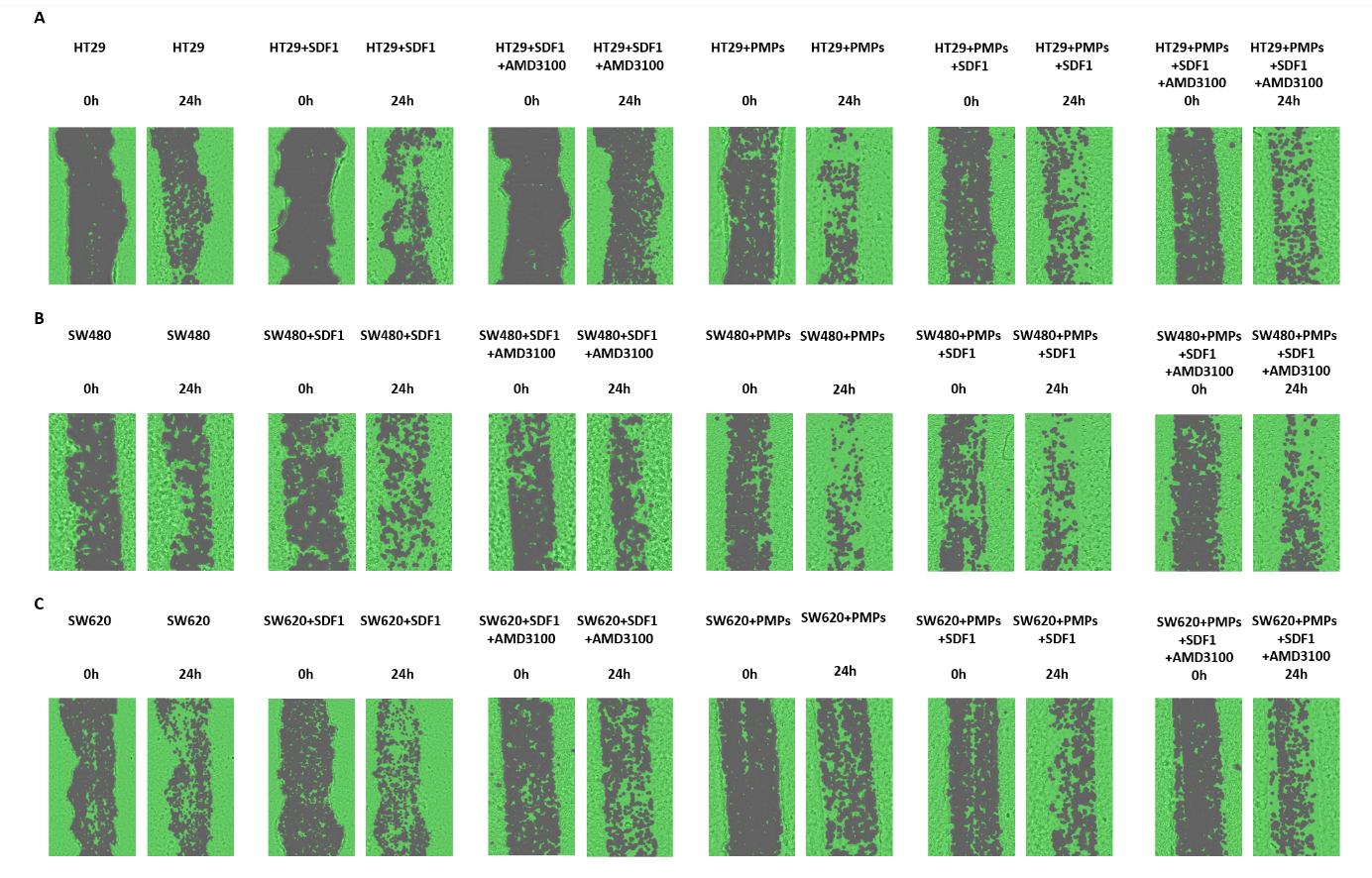
**

**Fig. 3S. Effect of PMP internalization on the CXCR4-dependent migration potential of CRC cells.** Representative images of wound closure of HT29 (A), SW480 (B) and SW620 (C) cells, control and preincubated for 4 h with PMPs in the presence or absence of SDF-1 and/or the CXCR4 antagonist AMD3100, after a 0h- and 24-hour migration period.


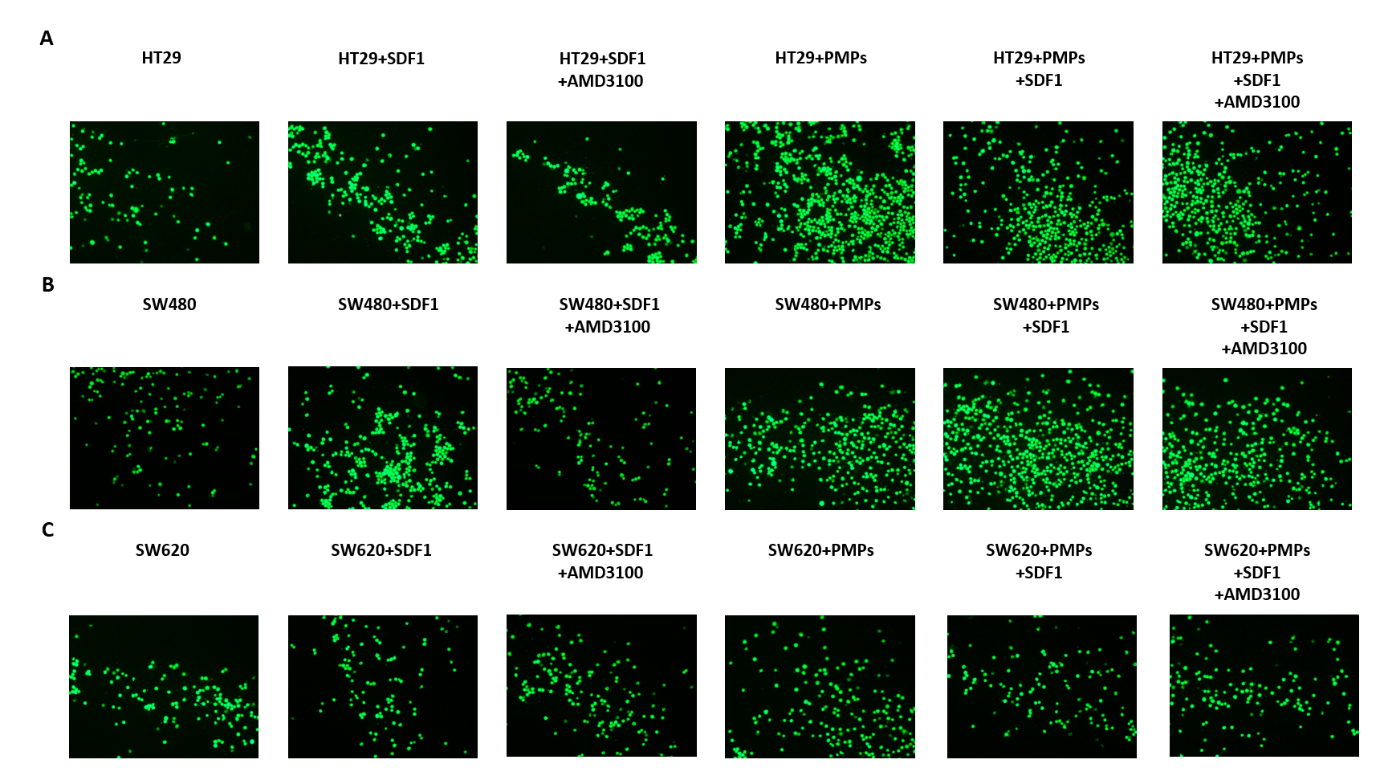


**Fig. 4S. Effect of PMP internalization on the CXCR4-dependent migration potential of CRC cells** **through un-coated Boyden chambers.** Representative images of CellTracker labelled HT29 (A), SW480 (B) and SW620 (C) cells, control and preincubated for 4 h with PMPs in the presence or absence of SDF-1 and/or the CXCR4 antagonist AMD3100, migrated through un-coated Boyden chambers.

**
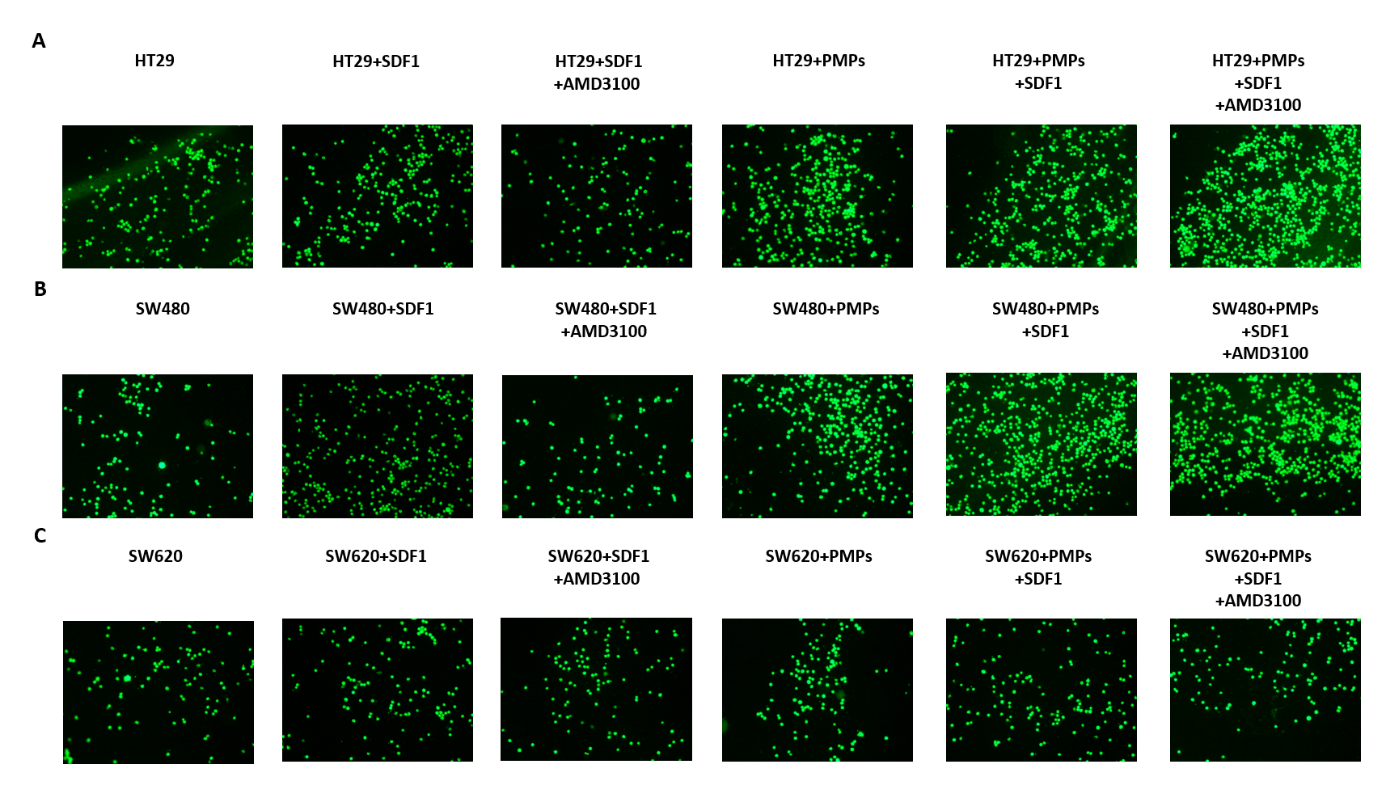
**

**Fig. 5S. Effect of PMP internalization on the CXCR4-dependent migration potential of CRC cells** **through Matrigel-coated Boyden chambers.** Representative images of CellTracker labelled HT29 (A), SW480 (B) and SW620 (C) cells, control and preincubated for 4 h with PMPs in the presence or absence of SDF-1 and/or the CXCR4 antagonist AMD3100, migrated through Matrigel-coated Boyden chambers.

**
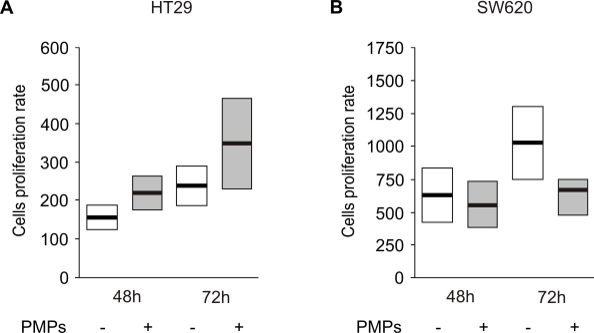
**

**Fig. 6S. Proliferation of HT29 and SW620 cells after the incorporation of PMPs.** Proliferation of HT29 (A) and SW620 (B) cells after 4 h of incubation with PMPs (at a dose of 50 µg of PMPs per 10^6^ cells and at a final concentration of 100 µg/ml) in appropriate medium not supplemented with FBS at 37 °C in a humidified atmosphere with 5% CO_2_. Quantified data (presented and statistically analysed as in Fig. 1) of cell proliferation for 48 and 72 h, calculated relative to cell proliferation for 0 h, N = 5.


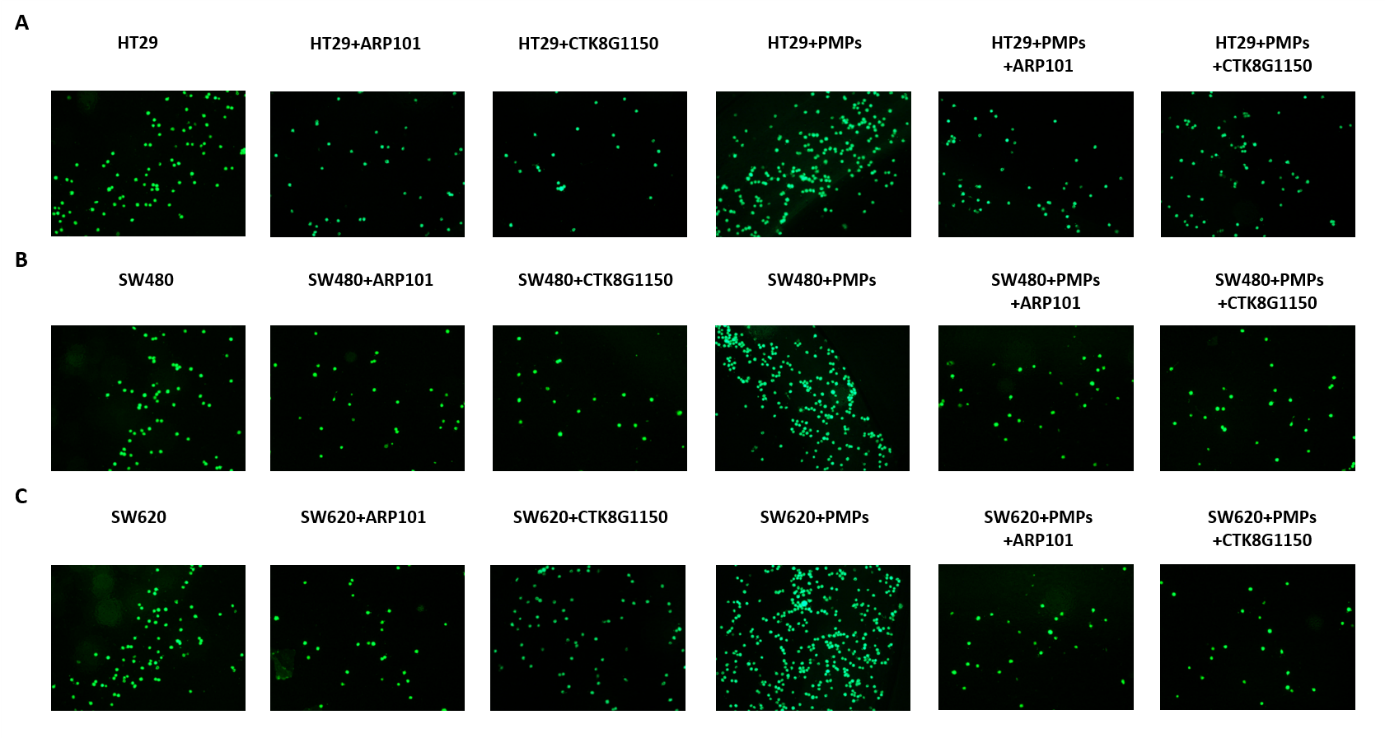


**Fig. 7S. Effect of PMP internalization on the invasive potential of CRC cells** **through gelatin-coated Boyden chambers in the presence of MMP-2 and MMP-9 inhibitors.** Representative images of CellTracker labelled HT29 (A), SW480 (B) and SW620 (C) cells, control and preincubated for 4 h with PMPs in the presence or absence of MMP-2 or MMP-9 inhibitors (ARP101 or CTK8G1150, respectively), migrated through gelatin-coated Boyden chambers.

**
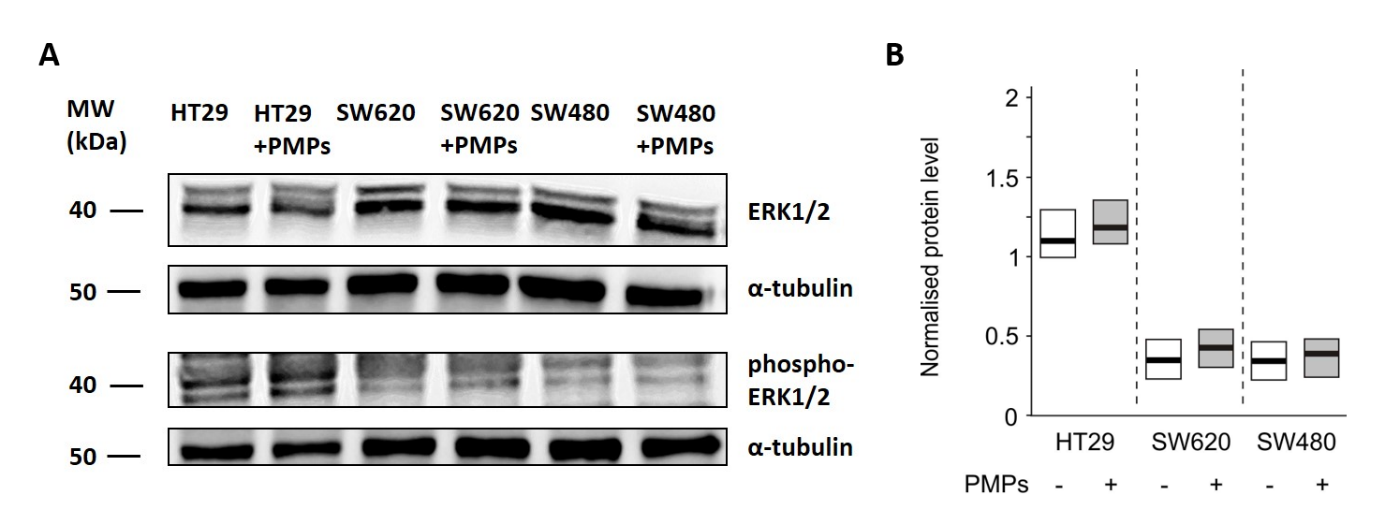
**

**Fig. 8S. Effect of PMPs on ERK1/2 phosphorylation.** A – Representative western blots of phosphorylated ERK1 (phospho-ERK1/2) and total ERK1/2 (ERK1/2) in CRC cells after 10 min of incubation with PMPs (at a dose of 50 µg of PMPs per 10^6^ cells and at a final concentration of 100 µg/ml) in appropriate medium not supplemented with FBS at 37 °C in a humidified atmosphere with 5% CO2. B – Normalized densitometric values of phosphorylated ERK1/2 (phospho-ERK1/2) and the total ERK1/2 (ERK1/2) ratio. Quantified data are presented and statistically analysed as in Fig. 1, N = 5.


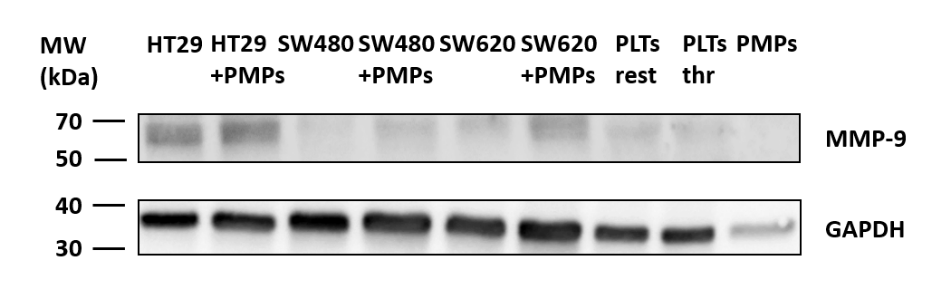


**Fig. 9S. MMP-9 expression in CRC cells stimulated with PMPs, in resting and activated platelets and in PMPs.** Representative western blots of MMP-9 expression in CRC cells (after 4 h of incubation with PMPs as described above), in platelets (PLTs) not activated and activated with 2 U/ml thrombin and in PMPs.

**
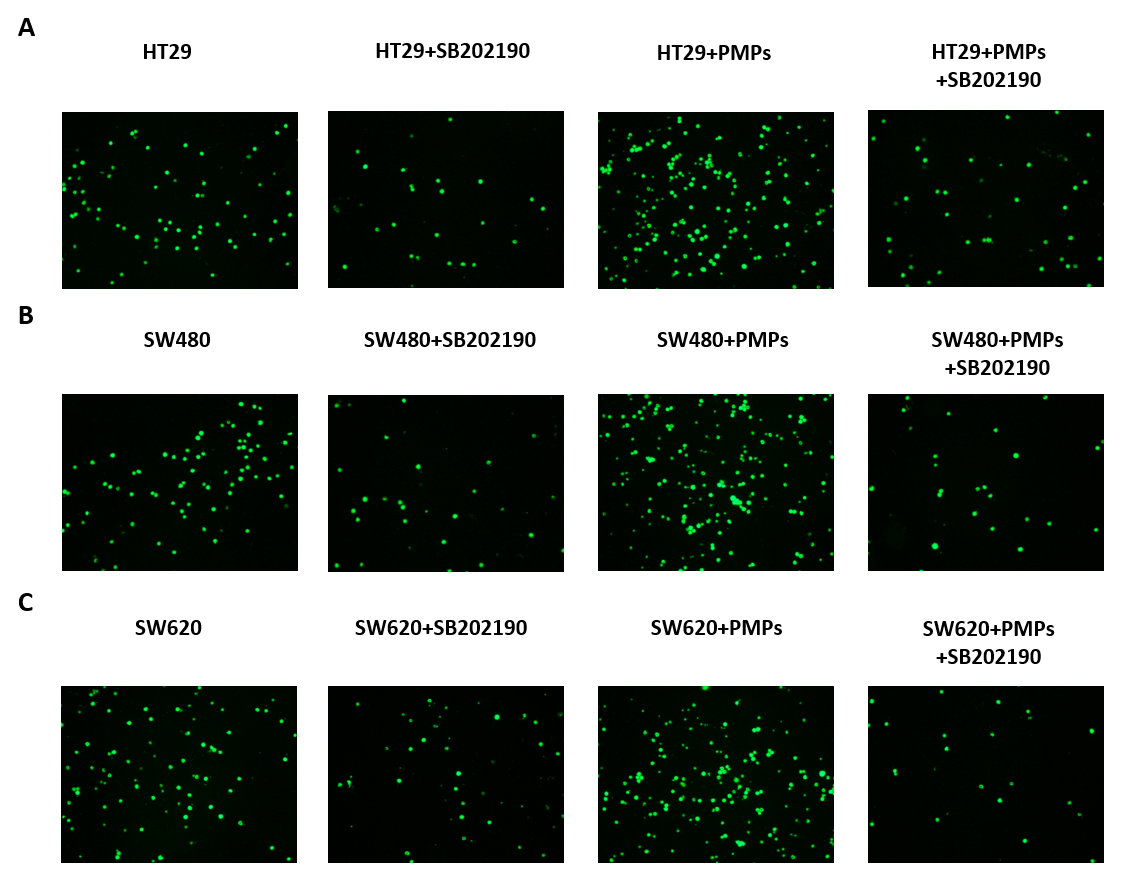
**

**Fig. 10S. Effect of PMP internalization on the invasive potential of CRC cells** **through gelatin-coated Boyden chambers in the presence of phospho-p38MAPK inhibitor.** Representative images of CellTracker labelled HT29 (A), SW480 (B) and SW620 (C) cells, control and preincubated for 4 h with PMPs in the presence or absence of phospho-p38MAPK inhibitor, SB202190, migrated through gelatin-coated Boyden chambers.

**
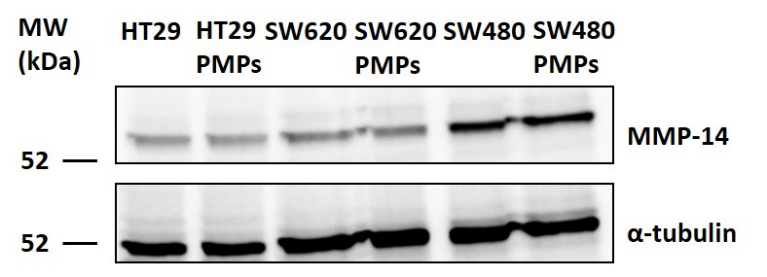
**

**Fig. 11S. Effect of PMPs on MMP-14 expression.** Representative western blots of MMP-14 expression in CRC cells after 4 h of incubation with PMPs (at a dose of 50 µg of PMPs per 10^6^ cells and at a final concentration of 100 µg/ml) in appropriate medium not supplemented with FBS at 37 °C in a humidified atmosphere with 5% CO2.
