# Supplementary material for: Platelet-derived microparticles stimulate the invasiveness of colorectal cancer cells via the p38MAPK-MMP-2/MMP-9 axis
Source: Cell Commun Signal. 2023 Mar 7;21:51. doi: 10.1186/s12964-023-01066-8 (PMC9990213; doi:10.1186/s12964-023-01066-8)
Supplement: Supplementary file 3 — Additional file 2: Supplementary Methods. [file 12964_2023_1066_MOESM3_ESM.docx]

**Supplementary Methods**

*Cell culture*

Human colorectal adenocarcinoma cell lines with different phenotypic migratory potentials, HT29 (epithelial), SW480 (mesenchymal) and SW620 (strongly mesenchymal), were purchased from the American Type Culture Collection (ATCC, Manassas, VA, USA). Ht29 cells were cultured in McCoy’s 5A medium (Thermo Fisher Scientific, Waltham, MA, USA), SW480 and SW620 cell were cultured in RPMI 1640 medium (with the ATCC modification, Thermo Fisher Scientific), all with the addition of 10% fetal bovine serum (FBS; Sigma–Aldrich), penicillin/streptomycin (pen/strep) (Thermo Fisher Scientific) and primocin (InvivoGen, San Diego, CA, USA). Cells were cultured at 37 ˚C in an incubator containing 5% CO2 and 95% humidified air. Cells were routinely tested for mycoplasma (PlasmoTest, InvivoGen).

*Isolation of PMPs*

PMPs were obtained from platelet concentrates purchased from Central Blood Donor in Lodz. Firstly, platelets were separated from plasma at 200 x g for 20 min at RT. Then after washing twice in PBS, platelets were stimulated with 2 U/ml thrombin and 2.5 mM CaCl2 for 20 min on rotary shaker. PMPs were obtained after centrifugation of stimulated platelets for 1500 x g for 20 min at RT followed by ultracentrifugation of resulting suspension at 100,000 x g for 2h at 4°C.

*Confocal imaging*

Prior to addition of platelet-derived microparticles to HT29 and SW620 cells, PMPs were labelled using PKH67 Fluorescent Cell Linker kit (Sigma Aldrich, St. Louis, MO, USA). Briefly, PMP obtained according to protocol described above, were incubated with PKH67 solution for 4 min. Unbound dye was removed by washing twice in PBS containing 1% BSA at 100,000 × g for 70 min at 4°C and pelleted PMP were resuspended in appropriate medium. HT29 and SW620 cells were plated in 8-well poly-lysine coated glass chamber slides, washed in PBS and incubated with PKH67-labeled PMPs (at the dose of 50 µg of PMPs per 10^6^ of cells and at the final concentration of 100 µg/ml) or with cell culture medium (control) for 4 h. Afterwards slides were fixed with 3% PFA at RT for 10 min followed by incubation with 0.1% Triton X-100 for 20 min at RT for cell permeabilisation. Excessive amount of PFA and Triton X-100 were removed by intermediate washing steps in PBS. Lastly, the particular cell components were labeled after addition of following dyes: Alexa-Fluor 594-conjugated Wheat germ agglutinin (5 µg/ml, plasma membrane labeling dye) and Hoechst 33342 (5 µg/ml cell-permeant nuclear dye). In another set of experiments, after incubation with PMPs, cells were labeled with PKH67 and washed twice in PBS containing 1% BSA to bound free dye. After cells fixation and permeabilisation, the antibodies against CXCR4 (10 µg per 10^6^ cells) or CD61 (10 µg per 10^6^ cells) were added followed by incubation with Hoechst 33342 (5 µg/ml). After removal the unbound antibodies and dyes by washing in PBS, cells on slides were visualized using confocal microscope (Nikon D-Eclispe C1) analyzed with EZ-C1 version 3.6 software.

*Wound healing assay*

HT29 and SW620 cells were seeded in 24-well plates and grew in medium appropriate for each cell line containing 10% FBS. At 90% confluence, medium was replaced with medium without FBS, containing PMPs (at the dose of 50 µg of PMPs per 10^6^ of cells and at the final concentration of 100 µg/ml of total protein as assayed using BCA method). For control wells, medium with 0% FBS and without PMPs was added. Cells were incubated for 4h under constant conditions 37 °C and 5% CO_2_. In order to block cell migration dependent on CXCR4, cells were incubated with a CXCR4 antagonist AMD3100 (at final concentration of 10 μM) or with anti-CXCR4 antibodies (at final concentration of 100 μg/ml). Afterwards, the scratch wound was made on a monolayer of cells adhered to the bottom of well surface, using a sterile pipette tip in each well. Following this step, the medium was removed, wells were washed twice with warm PBS and appropriate medium supplemented with 1% FBS and containing or not SDF-1 (at the final concentration of 400 ng/ml) was added. We optimized the low concentration of FBS in separate experiments in order to achieve appropriate conditions for cell migration without any significant effect of microvesicles present in FBS. Cell migration was evaluated by measuring the cell-free surface at the beginning of the experiment (immediately after the scratch was made, t0 ) and after each 2h till 24-hour period, using SPARK (Tecan) equipment supplemented with light camera and special chamber providing constant conditions of temperature and humidity. Images were analyzes in ImageJ Software (Fiji) with additional package for wound healing assay. Migration rate (recovery) was expressed as the percentage of wound closure, normalized to t0 recovery.

*Migration and invasion assays*HT29 and SW620 cells were seeded in 24-well plates and grew in medium appropriate for each cell line containing 10% FBS. At 90% confluence, cell were incubated with PMPs (at the dose of 50 µg of PMPs per 10^6^ of cells and at the final concentration of 100 µg/ml) in appropriate medium not supplemented with FBS for 4 h at 37°C in a humidified atmosphere with 5% CO2. For control wells, medium with 0% FBS and without PMPs was added. In order to block cell migration dependent on CXCR4, cells were incubated with a CXCR4 antagonist AMD3100 (at final concentration of 10 μM) or with anti-CXCR4 antibodies (at final concentration of 100 μg/ml). The migration and invasion properties of tumor cells after incorporation of PMPs were evaluated in uncoated or Matrigel-coated Boyden chambers; respectively. Briefly, filters (6.5 mm in diameter, 8 µm pore size) were left uncoated or were coated with either Matrigel (diluted 1:10 in PBS, final concentration 10 mg/ml, Thermofisher), for migration or invasion assays, respectively. Since matrigel solution is liquid at 4°C, but it gels very quickly at RT, thus Matrigel solution was thawed at 4 °C overnight and all the material (pipettes, tips, and forceps) were stored in the -20°C before the experiment. Porous membrane in the Boyden chamber was covered with Matrigel and left at 37°C for 30 min for gelling. The lower chambers contained appropriate medium supplemented with 1% BSA and with or without SDF-1 (at the final concentration of 400 ng/ml). Cells incubated with PMVs or not were detached from plate using accutase, suspended in appropriate medium not containing FBS, counted using crystal violet and incubated with CellTracker™ Green CMFDA Dye (Thermo Fisher Scientific) for 30 min at 37°C, 5 % CO2. After removing the unbound dye by centrifugation (180×g, 5 min, RT), cells suspended in appropriate medium not containing FBS were loaded onto the upper compartments (2 × 105 cells/chamber) and left at 37°C, 5% CO2 for 3 hours to migrate through uncoated (migration) or Matrigel-coated (invasion) filters. After washing in PBS, cells that passed through to the filter were evaluated on the undersides of filters using a Nikon inverted microscope (Eclipse E600; Nikon, Tokyo, Japan) at 10× magnification. Cell migration across the membrane was quantified in ten fields of view for each membrane.

*Immunoblotting*CRC cells were incubated for 4 h with PMPs as described above for the detection of CXCR4, MMP-2, and MMP-9 and lysed using RIPA buffer (Sigma Aldrich) supplemented with protease inhibitor cocktail (dilution 1:100, Thermo Fisher Scientific) or incubated for 10 min with PMPs for the detection of nonphosphorylated and phosphorylated ERK1/2 and p38MAPK and lysed with M-PER Mammalian Protein Extraction Reagent (Thermo Fisher Scientific) supplemented with protease inhibitor cocktail (dilution 1:100, Thermo Fisher Scientific Aldrich) and phosphatase inhibitor cocktail (dilution 1:100, Thermo Fisher Scientific). Cell were lysed for 30 min on ice. Samples were centrifuged at 20,000 g for 20 min at 4°C to remove cellular debris and protein concentration in lysates was determined according to BCA method (Pierce BCA Protein Assay; Thermo Fisher Scientific). Samples (25 µg of protein) were incubated with 1x sample buffer containing β-mercaptoethanol (at final concentration of 1%, ) for 5 min at 95°C and separated on SDS-PAGE 10% polyacrylamide gel at constant voltage of 150 V and transferred on nitrocellulose membrane using Turbo blotting equipment (Bio-Rad). After blocking of non-specific antibodies binding in Tris-buffered saline/ 0.1% Tween-20 (TBST), containing 5% nonfat dry milk or 5% BSA/0.1% TBST, membrane was incubated with mouse rabbit anti-human antibodies against nonphosphorylated p38MAPK (Cell Signaling, 1:1000 in 5% BSA/TBST) and ERK1/2 (Thermo Fisher Scientific, 1:5000 in 5% BSA/TBST) and phosphorylated p38MAPK (Cell Signaling, 1:1000 in 5% BSA/TBST) or mouse anti-human antibodies against CXCR4 (clone 12G5, Thermo Fisher Scientific, 1:5000 in 5% milk/TBST), MMP-2, MMP-9 (Thermo Fisher Scientific, 1:1000 in 5% milk/TBST) and phosphorylated ERK1/2 (Thermo Fisher Scientific, 1:5000 in 5% BSA/TBST) overnight at 4°C, followed by incubation with secondary goat anti-mouse (Santa Cruz Biotechnology, diluted 1:2500 in TBST) or goat-anti-rabbit IgG (Thermo Fisher Scientific, diluted 1:5000 in TBST), conjugated with horseradish peroxidase (HRP) for 2h at RT. Unbound antibodies were removed by intermediate washing steps in TBST. Antibodies against GAPDH or α-tubulin (1: 20000, Santa Cruz Biotechnology) were used to verify equal loading. Protein bands were visualized by exposure onto Hyperfilm ECL chemiluminescence film in a BioMax Cassette (Kodak) and development on an M35 X-OMAT processor (Kodak).

*Flow cytometry*

CRC cells were seeded in 24-well plates and grown in culture medium. At 90% confluence, the medium was replaced with medium without FBS, containing or not containing (control) PMPs. The cells were incubated for 4 h at 37 °C in a humidified atmosphere with 5% CO_2_. Accutase (Sigma Aldrich) was used instead of routinely used trypsin for cells detaching since accutase rather than trypsin is recommended for flow cytometric measurements of surface antigens. For labeling with antibodies, 10^6^ cells per milliliter were applied. Cell were incubated with 10 µg of anti-CXCR4 PE-conjugated antibodies (Thermo Fisher Scientific) or FITC or PE-conjugated antibodies (BE Bioscience) against the integrin subunits: α2, α6, αv, Ib, β3, β4, αIIβ or glycoprotein Ib for 1h at RT. Samples were washed twice in PBS containing 1% BSA in order to remove resting and unbound antibodies and fixed with 1% Cellfix (BD Biosciences) for 1h at RT. For intracellular detection of CXCR4, before the addition of antibodies, cells were first fixed with 1% Cellfix for 30 minutes RT in dark and permeabilisation of cell membranes was made by addition of 1% Tween 20 for 20 minutes RT. Flow cytometric measurements were performed using a LSR II system (BD Biosciences) and data were analysed using FACS Diva ver. 6.0 (BD Bioscience). At least 10,000 cells were analyzed per sample. All results were processed using FACS/Diva ver. 6.0 software (Becton-Dickinson). Appropriate gates were applied to select cancer cell populations in forward, side scatter and fluorescence plots. The specific fluorescence-positive cells were evaluated after subtracting non-specific isotype IgG binding (by setting gate for isotype control to 1% of positive cells).

*MMP2 and MMP-9 activity assays*We set other experiments to determine the activity of cellular MMP. In one of them, we used FITC-labeled gelatin which, upon gelatinolytic activity, is converted into bright fluorescent peptides. Firstly, 96-well black plate was coated with 0.1 mg/ml of FITC-gelatin for 48h at 4°C in dark. After removal of unbound gelatin, 50×10^3^ cells per ml, pre-incubated for 4h at 37 °C and 5% CO2 with PMPs (at the dose of 50 µg of PMPs per 10^6^ of cells and at the final concentration of 100 µg/ml) were added to gelatin-coated wells (0.1 mg/ml) and incubated for 24h. The activities of MMP-2 and MMP-9 were blocked by the appropriate inhibitors of MMP-2 (ARP101) or MMP-9 (CTK8G1150) at the final concentrations of 20 µM. The appearance of green fluorescence was measured at 520 nm on microplate reader (Bio-Rad). In another set of experiments, we measure the activity of MMP-2 and MMP-9 by assaying the migration of CRC cells previously incubated with PMPs as described above through 0.2 % gelatin-coated filters in the Boyden chambers in the procedure similar to that described for migration/invasion assay. The activities of MMP-2 and MMP-9 were blocked by the appropriate inhibitors MMP-2 (ARP101) or MMP-9 (CTK8G1150) at the final concentrations of 20 µM. Moreover, to verify whether the effect of PMPs on the phosphorylation of p38MAPK can affect the gelatinolytic activities of CRC cells, the specific inhibitor of p38MAPK, SB202190 was applied at the concentration of 5 µM in both assays described above.

*Enzyme-linked immunosorbent assay for MMP-9*HT29, SW480 and SW630 cells were incubated with PMPs as described above, and the MMP-9 concentration was evaluated in the undiluted conditioned medium using ELISA according to the manufacturer’s instructions (Biotechne). Briefly, medium was incubated with MMP-9 antibodies pre-coated on microplate for 2 h RT on orbital shaker. After washing away any unbound substances, an enzyme-linked polyclonal antibodies specific for MMP-9 were added to the wells and following a wash to remove any unbound antibody-enzyme reagent, a substrate solution was added to the wells and colour developed in proportion to the amount of MMP-9 bound in the initial step. The colour development was stopped and the intensity of the colour was measured at 450 nm. The concentration of MMP-9 was calculated according to the protein concentration in CRC cells lysates.

*Statistical analysis*Data are presented as mean, standard error and min-max values or as median and interquartile range (IRQ, lower [25%] quartile to upper [75%] quartile, depending on data scale and distribution. The normality of the data distributions was verified with the Shapiro-Wilk test, variance homogeneity was tested with Levene’s test. The Mann-Whitney U-test was employed to evaluate the significance of differences between two independent groups of variables departing from normality. Otherwise, Student’s t-test for independent samples was used for comparing two groups. One-way ANOVA (with relevant post hoc Tukey’s multiple comparisons test) was used to compare multiple data sets, while the Kruskal-Wallis test followed by the post hoc all-pairwise comparisons Conover-Inman test was used for data not satisfying the criterion of normality and/or variance homogeneity. Statistical analysis was performed using Statistica v. 12.5.
